# The Delineation of Advanced‐Level Practice Within UK District Nursing: A Cross‐Sectional Comparative Study Before and After Policy Implementation

**DOI:** 10.1111/inr.70091

**Published:** 2025-08-05

**Authors:** John Unsworth, Crystal Oldman, Joanne Atkinson, Dania Comparcini, Valentina Simonetti, Giancarlo Cicolini, Kristina Mikkonen, Marco Tomietto

**Affiliations:** ^1^ Department of Nursing, Midwifery and Health, Faculty of Health and Life Sciences Northumbria University Newcastle upon Tyne UK; ^2^ Section of Nursing, Department of Precision and Regenerative Medicine and Ionian Area University of Bari ‘Aldo Moro’ Bari Italy; ^3^ The Queen's Nursing Institute London UK; ^4^ Department of Social Work, Education and Community Wellbeing, Faculty of Health and Life Sciences Northumbria University Newcastle upon Tyne UK; ^5^ Section of Nursing, Interdisciplinary Department of Medicine Aldo Moro’ University of Bari Bari Italy; ^6^ Department of Innovative Technologies in Medicine and Dentistry G.d'Annunzio’ University of Chieti Pescara Italy; ^7^ Research Unit of Health Science and Technology University of Oulu Oulu Finland; ^8^ Medical Research Center Oulu Oulu University Hospital and University of Oulu Oulu Finland

**Keywords:** Advanced practice nursing, district nursing, nursing roles, United Kingdom, workforce

## Abstract

**Introduction:**

Advanced practice nursing is key in response to increasing healthcare demands and the need for Universal Health Coverage. District nursing, which involves care for people in their own homes, has undergone a significant transformation to manage complex long‐term conditions and reduce hospital admissions.

**Aim:**

This study aims to identify the advanced practice domains of UK district nurse team leaders, before and after policy reforms designed to prevent unnecessary admission to hospital. A secondary aim is to compare the advanced practice domains of district nurse team leaders with those of advanced practice nurses working in family and community nursing.

**Methods:**

A cross‐sectional study was conducted. Data were collected via an online survey using the Family and Community Nursing Advanced Practice Scale, assessing five key domains of advanced practice. The study employed ANOVA and *t*‐tests to compare groups.

**Results:**

A total of 448 district nurse team leaders participated in this study, and secondary data from 548 family and community nurses were retrieved. No significant differences were found across qualification groups for most advanced practice domains, except for medication prescribing. Comparisons with advanced practice nurses in family and community settings revealed significantly higher scores in all domains and similar scores in leadership competencies.

**Discussion:**

Experiential learning plays a crucial role in developing advanced practice competencies beyond formal qualifications. The results align with Kolb's theory, highlighting the importance of clinical exposure and mentorship in shaping advanced nursing skills.

**Conclusion:**

District nurse team leaders exhibit key advanced practice competencies. Credentialing frameworks may help formally recognise the expertise developed through clinical experience.

**Implications for nursing and health policy:**

Policymakers should consider enhancing experiential learning opportunities and credentialing systems to support district nurses in their transition to advanced practice.

## 1 Introduction

Advanced practice nursing has grown exponentially across the world in response to increasing demand and the need to provide timely access to services and Universal Health Coverage (Lopez‐Junior 2021). In many countries, individuals who are registered nurses, or midwives can complete master's level study to become either an advanced nurse practitioner, a clinical nurse specialist, nurse anaesthetist or an advanced midwife. While there is wide variation in job titles and systems of regulation, the International Council of Nurses (2020) defines an advanced practice nurse (APN) as either a specialist or generalist who, through master's education, has acquired expert knowledge and the competencies required to make complex decisions and perform as a nurse at an advanced level. According to the International Council of Nurses (2020), APNs operate beyond the scope of registered nurses. They may be generalists or specialists, capable of managing full episodes of care with greater autonomy. Their role includes advanced judgement, decision‐making, and diagnostic reasoning, along with the authority to diagnose, prescribe, order tests, refer, and manage admissions and discharges.

In most health systems, APNs work alongside other colleagues to deliver care to patients either in a nursing team or as part of a wider multidisciplinary team (Kilpatrick et al. 2023). The wholesale change of a workforce and its role to advanced practice nursing is relatively rare, and this paper explores one of the role changes driven by policy drivers around managing complex chronic disease and reducing unnecessary emergency admissions to hospital. The paper specifically delineates advanced‐level practice among this workforce following policy implementation.

Several policy issues drove the development of district nursing roles towards advanced nursing practice. These included the need to manage complex chronic diseases and prevent unnecessary emergency hospital admissions. Globally rising rates of chronic illnesses, such as cardiovascular disease, diabetes, and respiratory problems, are placing an increasing burden on health services (Sleeman et al. 2019). Many of these diseases are characterised by exacerbations requiring medical intervention and often necessitating an emergency admission to hospital (Nuffield Trust 2024). Across Europe, the pressure of emergency admissions and issues around patient flow through hospitals and back into the community are challenging health system resilience (European Union 2023). The United Kingdom has experienced problems for several years, with the winter of 2024–2025 being particularly bad because of a mixture of increased admissions combined with increased rates of respiratory infections (Ameneshoa et al. 2024).

The 2010 policy reforms arose out of concerns that a small number of patients with chronic diseases and complex needs had frequent admissions to the hospital. In addition, there was increasing recognition that a proportion of emergency admissions can be prevented through ambulatory care (Loyd et al. 2023; Sarmento et al. 2020; Dixon et al. 2012). A proportion of these so‐called ambulatory sensitive conditions can be safely and effectively managed either through day unit admission or in a community setting (Hodgson et al. 2019; Longman et al. 2015).

Recognising the potential to avoid unnecessary emergency admission, the National Health Service in England started to use re‐admission risk stratification tools from 2010 onwards (Kingston et al. 2020). These risk stratification tools identified people with complex needs who were at risk of emergency admission, allowing community nurses to proactively manage their care to prevent unnecessary admission to hospital (NHS England 2017). The primary objectives of the community matron policy were to improve the health and well‐being of individuals with long‐term conditions, prevent avoidable hospital admissions, and promote self‐management of health conditions. They achieve this by providing advanced nursing care, case management, and proactive support to patients in their homes and communities.

Case management initially followed a joint health and social care model (Lyon, Miller, and Pine 2006). It later evolved into a national policy initiative with the introduction of specialist community matron nursing roles (Drennan et al. 2011). These community matron roles were filled by nurses who were practising as APNs in order that they could assess patients, physically examine them, diagnose problems, prescribe medicines and other interventions, and refer to other services as necessary. Community matrons were usually existing community nurses who received additional education at postgraduate level to help them manage long‐term conditions such as chronic obstructive pulmonary disease. They were also educated to enable them to work as independent prescribers. However, in their evaluation of the community matron policy rollout, Drennan et al. (2011) identified that the policy was unevenly implemented because of financial constraints. Many areas sought to develop the roles of existing staff, especially in district nurse team leaders (Dickinson, Gough, and Bain 2011). This paper explores the advanced practice competencies in this area of nursing care, by adopting a validated tool for evaluating those competencies and by considering the main policy reforms.

## 2 Background

### 2.1 The Evolution of District Nursing Towards Advanced Practice Nursing

District nursing is a specialised field of community nursing providing care at home for individuals and their families. It is one of the very first nursing specialisms, having been organised in 1887 with formal training and certification from 1891 (Fox 1994). District nursing operates in many countries across the world including New Zealand, Australia, Canada, Norway, the Netherlands, and Sweden. In other countries, it is called home nursing or community health nursing (Verheij and Kerkstra 1992).

McBride et al. (2024: 2) define district nursing as ‘a specialized field within community nursing that focuses on providing comprehensive care at an advanced level to individuals with complex health needs’. The scope of district nursing practice is wide, encompassing the proactive management of long‐term conditions, palliative and end‐of‐life care to the provision of complex wound care and the care of people with physical health problems in their own homes.

Several studies have highlighted how increasing complexity in home‐based care requires a review of the competencies for nurses in this field (Rusli et al. 2022; Raleigh and Allan 2017). These include advanced competencies related to clinical reasoning and decision‐making because of the levels of autonomy that community practice brings (Rusli et al. 2022).

In the United Kingdom, district nursing team leaders oversee teams that deliver home‐based care. These teams typically include registered nurses and healthcare assistants working collaboratively to support patients. Most district nurse team leaders undertake an approved course (NMC 2022) to prepare them for their role. The course is at a postgraduate level, either a full master's degree or a postgraduate diploma, and it includes independent prescribing. The course is not mandatory, and there are examples of district nurse team leaders without this qualification. Those without the qualification often undertake continuing professional development modules at postgraduate level to enable them to manage complex patients and to prescribe independently.

In line with changes in policy, the professional standards for district nursing courses have continually changed over time. In 2015, the Queen's Nursing Institute (QNI and QNIS 2015) developed standards for district nursing education, which were built around the four pillars of advanced practice (Health Education England 2017). These included standards for clinical care, including history taking, physical examination, diagnosis, and prescribing alongside standards for leadership, education, and research. At the same time, universities started to use these standards to develop their approved courses, moving them to master's level qualifications. The standards for district nursing were updated in 2023, further reinforcing advanced practice nursing and the core competencies for an advanced practice nurse (QNI 2023). In addition to changes to formal district nursing courses, continuing professional development around advanced clinical skills, diagnosis, and prescribing was offered to the existing workforce from 2010 onwards (Bain and Moggach 2019; Aldridge‐Bent 2011; Baid et al. 2009). These continuing professional development courses were designed to upskill the existing workforce who may have completed their district nursing qualification prior to 2015.

### 2.2 District Nurses as Clinical Nurse Specialists

The International Council of Nurses (ICN) has identified that the most common types of APNs are clinical nurse specialists (CNS) and nurse practitioners (ICN 2020). Irrespective of the type of advanced practice nurse, they are usually prepared to master's level, have designed roles around the provision of direct or indirect care and complex health problems, integrate research into practice, have extended autonomy and advanced judgement, decision‐making, and diagnostic reasoning skills (ICN 2020). In addition, in many jurisdictions, APNs have the authority to diagnose, prescribe, order, and interpret diagnostic tests and refer patients to others (ICN 2020). District nursing in the United Kingdom is most closely aligned with the ICN definition of a CNS. The ICN (2020) describes how for a CNS, the specialism may be defined by the location of care delivery or a specific condition. Furthermore, a CNS differs from a specialist nurse in so much as they take extended responsibility for healthcare and service improvement, education, and leadership while at the same time delivering direct and indirect care. Crucially, a CNS usually works with patients with an established diagnosis rather than with undifferentiated health problems (ICN 2020).

As a team leader, a district nurse takes responsibility for complex care and manages changes in the patient's established condition, e.g., exacerbation of a chronic disease. They are trained to assess, diagnose, and prescribe medicines to manage such established conditions alongside leading and guiding a larger team of nurses who deliver care to the population. The specialism for district nurses is community practice and home‐based care. Around half of district nursing team leaders spend less than 40% of their time in direct and indirect care, with the remaining time devoted to leadership, education, and service improvement (QNI 2024). This is a similar profile to that described by the ICN as a CNS (ICN 2020).

### 2.3 Role Delineation of APNs

Globally, several studies have been conducted to differentiate advanced practice nursing roles from other roles (Gardner et al. 2013; Gardner et al. 2016; Jokiniemi et al. 2022a). Most of these studies have been conducted among hospital‐based APNs using the Advanced Practice Role Delineation Tool (Mick and Ackerman 2000) developed from the Strong model of advanced practice (Ackerman et al. 1996).

Several studies have sought to delineate advanced practice nursing roles from registered nurses (Jokiniemi et al. 2022a, 2022b; Nahari et al. 2024), and these all suggest that the role of an APN is significantly different from that of a registered nurse across four of the five domains of advanced practice nursing. Only direct care scores lower for CNSs practising in Finland in the study by Jokiniemi et al. (2022b), where the CNS scores 0.72 (out of 4.00) compared with the registered nurse who scores 2.60. A study in Australia conducted by Gardner et al. (2016) suggested that CNSs scored lower for direct care (2.74) when compared with nurse practitioners (3.47), which confirms the earlier exploratory work by Mick and Ackerman (2000), where practitioners were asked to rank the relevant importance to their role of different aspects of practice. Nahari et al. (2024) suggest that APNs score higher for direct care (3.57) than registered nurses (2.96) on direct care in their study in Saudi Arabia.

One of the most comprehensive role delineation studies was conducted in New Zealand (Carryer et al. 2018). Data were collected from 3,255 nurses using the Advanced Practice Role Delineation Tool. Data from hundreds of nursing positions were collected, including from 44 district nurses. The most common roles were nurse practitioner, CNS, domain nurse, and registered nurse. The nurse practitioners and clinical nurse specialists had similar scores across the five domains, with nurse practitioners scoring slightly higher for direct care and leadership than their clinical nurse specialist counterparts. In this study, district nurses in New Zealand had similar scores for direct care but lower scores for support for systems, education, research, and leadership. Except for this study, there is a dearth of evidence comparing the role of the district nurse with other types of APNs.

## 3 Aim of the Study

This study aims to examine the scope of advanced practice of UK district nurse team leaders, comparing those who do not have an advanced practice qualification with those who completed their qualification before and after the 2010 policy reforms.

Furthermore, a secondary aim of this study is to compare the scope of advanced practice of district nurse team leaders with established APNs working in family practice and community nursing.

## 4 Methods

### 4.1 Research Design

This study utilised a cross‐sectional design and adhered to the Strengthening the Reporting of Observational Studies in Epidemiology (STROBE) guidelines (von Elm et al. 2008) for reporting.

### 4.2 Sample and Setting

The target population for this study included district nurse team leaders in the United Kingdom. Eligibility criteria required participants to be engaged in clinical practice at the time of data collection. The sample size calculation was conducted using G*Power (Faul et al. 2007) to ensure adequate power for comparing three groups (no advanced qualification, qualification obtained before 2010, and qualification obtained after 2010). Assuming an effect size of 0.50, a significance level (α) of 0.05, and a statistical power of 0.80, the calculation indicated a minimum of 42 participants per group would be required.

To test the secondary aim, the data collected in this study were compared to a dataset of APNs working in family practice or community roles from a previous study (Unsworth et al. 2025), and a secondary data analysis was performed.

### 4.3 Data Collection

Data collection employed a convenience sampling approach. An online survey was developed using the Joint Information Systems Committee survey platform, a data technology provider for UK higher education. To maximise participation and maintain engagement, periodic reminders were sent via professional channels, including emails to network members and promotion through social media and newsletters. Data collection occurred between September and December 2024.

The Family and Community Nursing Advanced Practice scale (FCN‐APS), comprising 27 items, was designed with a 5‐point Likert response format for each item, ranging from 5 ‘very great extent’, 4 ‘great extent’, 3 ‘some extent’, 2 ‘little extent’, to 1 ‘not at all’. The scale assessed five factors of advanced practice competencies: clinical care (7 items), leading practice (7 items), clinical reasoning (5 items), health promotion (5 items), and ethics (3 items). These domains were initially generated from Hamric's model (Hamric et al. 1996), the Nursing and Midwifery Board for Ireland model (Nursing and Midwifery Board of Ireland 2017), and the ESCO model (European Commission 2017). Content validity was ensured in previous studies by involving a panel of 10 clinical APNs from family (general) practice and community services. In detail, the content validity index was calculated at the scale level (S‐CVI), and it demonstrated a value of 0.98 for relevance and 0.99 for clarity. Construct validity for the scale was also established in previous studies, demonstrating acceptable fit indices (root mean square error of approximation (RMSEA) = 0.076, standardised root mean residual (SRMR) = 0.058, comparative fit index (CFI) = 0.891, and Tucker–Lewis index (TLI) = 0.879) and reliability scores (Cronbach's α = 0.920 for the entire scale, with factor‐specific α values ranging from 0.830 to 0.910) (Unsworth et al. 2025). The scale was developed and tested in the same professional, cultural, and organisational contexts where the current study took place. In this vein, no additional pilot tests or adaptations were required.

Secondary data were collected from UK‐based APNs working in family practice or community roles as part of the scale development process described by Unsworth et al. (2025).

### 4.4 Ethical Considerations

The data collection and analysis procedures were designed to ensure confidentiality and adhere to both national and European regulations, including the General Data Protection Regulation (European Union 2016) and the UK Data Protection Act (UK Government 2018). Electronic data were securely stored in a password‐protected folder, with access restricted to the principal investigator and designated members of the research team. Participants were provided with a detailed disclaimer on the initial survey page, outlining the study objectives and data handling procedures. By submitting the survey, participants indicated their informed consent to participate in the study. Ethical approval for the research was granted by Northumbria University (ref: 7020, 19 April 2024).

### 4.5 Data Analysis

The data were analysed using IBM SPSS v28.0 (IBM Corp. 2021) for descriptive statistics and groups’ comparison (ANOVA and *t*‐test) by accepting a statistically significant *p* value <0.05. Stata v13 (StataCorp 2013) was used to test the scale's validity by performing a confirmatory factor analysis (CFA).

As the primary aim of the study, an ANOVA comparison was employed between the three advanced practitioner qualification groups described above. The secondary aim was tested by employing a *t*‐test comparing district and community nurse leaders and family and community nurses. The effect size (Cohen's *d*) was estimated for both the ANOVA analysis, by comparing the group without an advanced qualification against the group with an advanced qualification certified before 2010, and the group with an advanced qualification certified from 2010 onwards, and the *t*‐test, by comparing district and community nurse leaders and family and community nurses.

The reliability of the scale in this study was assessed using Cronbach's α and McDonald's ω. For Cronbach's α, values greater than 0.90 are classified as excellent, values between 0.70 and 0.90 as good, values between 0.60 and 0.70 as acceptable, and values below 0.60 as unacceptable (DeVellis and Thorpe 2021). McDonald's ω is recommended as a complementary measure to Cronbach's α, as it provides a more robust estimate of reliability. McDonald's ω values greater than 0.60 are deemed acceptable, although some authors advocate for a threshold of 0.80 or higher (Mikkonen et al. 2022). Construct validity was evaluated through CFA, with model fit indices calculated to determine the adequacy of the scale. Acceptable thresholds for model fit are defined as RMSEA and SRMR values less than 0.08, and CFI and TLI values greater than 0.90 (Kline 2023).

## 5 Results

### 5.1 Sample Description

The sample included 448 participants, with a mean age of 45.92 ± 9.57 years (min = 25, max = 68). The majority were female (96.9%) and commenced their roles as district nurse team leaders after 2010 (90.4%). Regarding the educational level, 45.8% held an undergraduate degree as their highest qualification, while 50.7% had a postgraduate degree. Most participants were employed full‐time (82.4%), with 58.3% spending more than four days per week in clinical practice. Regarding the advanced practice qualification, 62.7% had completed their qualification in 2010 or later, 13.4% before 2010, and 23.6% declared no advanced practice qualification. Table 1 reports the sample's characteristics in detail. When considering the year of advanced practice qualification, the sample characteristics differed across the three groups described above: the highest educational qualification held among those with no advanced practice qualification was the diploma level, while that with the advanced practice qualification was predominantly at the postgraduate level (χ^2^ = 95.058, *p* < 0.001). Similarly, those with an advanced practice qualification also held a prescribing qualification (χ^2^ = 178.133, *p* < 0.001); no statistical difference was found regarding the exposure to clinical practice. Supplementary Table S1 presents in detail the main differences across the three groups. The secondary data from the APNs working in family and community nursing consisted of 548 participants, and the completion of the advanced practice qualification occurred after 2010 for 85.4% of the sample, and all the participants held the advanced practice qualification. The description of this sample is presented in detail in Unsworth et al. (2025). In this study, APNs working in family and community nursing demonstrated older age (50.12 ± 8.53) compared to district nursing team leaders (45.92 ± 9.57) (*t* = −7.316, *p* < 0.001) and statistically significant higher exposure to clinical practice (χ^2^ = 18.394, *p* < 0.001) (Supplementary Table S2).

**TABLE 1:** Sample characteristics.

Characteristic	*N*	Mean ± *SD*, Min–Max
Age (Mean ± *SD*, Min–Max)	448	45.92 ± 9.57 (25–68)
**Gender**	**N**	**%**
Female	434	96.9
Male	14	3.1
**Year commenced as DN team leader**	**N**	**%**
< 2010	43	9.6
≥ 2010	405	90.4
**Year completed advanced practitioner qualification**	**N**	**%**
No qualification	107	23.9
<2010	60	13.4
≥ 2010	281	62.7
Year qualified as registered nurse		
<2010	240	53.6
≥ 2010	208	46.2
**Highest qualification**	**N**	**%**
Bachelor degree (BSc Hons, BA Hons, BN, etc.)	205	45.8
Postgraduate diploma	123	27.5
Master's degree (MSc, MA, LLM, MRes, etc.)	71	15.8
Postgraduate certificate	33	7.4
Diploma in higher education	14	3.1
General education (GCSE, ordinary level, A level)	2	0.4
**Prescribing qualification**	**N**	**%**
Yes	396	88.4
No	52	11.6
**Type of work**		
Full‐time	369	82.4
Part‐time	79	17.6
**Days per week in clinical practice**		
1	56	12.5
2	46	10.3
3	85	19.0
4	113	25.2
5	124	27.7
>5	24	5.4

### 5.2 Scale Factors and Validation

Descriptive statistics and reliability analyses showed high internal consistency across the five factors, with Cronbach's α ranging from 0.821 to 0.908 among factors, and equal to 0.935 for the overall scale. Similarly, McDonald's ω ranged from 0.831 to 0.916 among factors and showed a value of 0.933 for the overall scale. Table 2 presents the detailed information for each factor.

**TABLE 2:** Descriptive statistics and reliability of the Family and Community Nursing – Advanced Practice Scale (FCN‐APS).

Factor/Scale	Mean ± *SD*	Min – Max	Cronbach's α	McDonald's ω
F1: Clinical Care	4.69 ± 0.51	2.29 – 5.0	0.907	0.916
F2: Leading Practice	4.29 ± 0.61	2.0 – 5.0	0.821	0.831
F3: Clinical Reasoning	3.29 ± 0.98	1.0 – 5.0	0.872	0.873
F4: Health Promotion	4.32 ± 0.73	2.0 – 5.0	0.908	0.910
F5: Ethics	4.47 ± 0.67	2.0 – 5.0	0.904	0.904
Overall Scale	4.23 ± 0.53	2.11 – 5.0	0.935	0.933

CFA results indicated acceptable model fit, with an RMSEA of 0.084, SRMR of 0.070, a CFI of 0.887 and a TLI of 0.874.

### 5.3 District Nurse Team Leaders and Advanced Practitioner Qualification

Analysis of variance revealed no significant differences across groups based on advanced qualification status (no qualification, qualified before 2010, and qualified in 2010 or later) for most factors and items. A single item in the factor ‘Clinical Reasoning’ and in detail ‘Prescribe medication’ showed statistically significant differences, with mean scores of 2.79 (±1.58), 3.35 (±1.49), and 3.16 (±1.34), respectively (*F* = 3.794, *p* = 0.023). Cohen's *d* values showed a negligible effect among factors. However, when considering the single items, several of them showed small/medium effects in favour of the groups holding an advanced qualification, and a few of them showed a negligible or small effect in favour of the group without an advanced qualification. Table 3 shows the detailed statistics for all the items of the scale.

**TABLE 3:** ANOVA between the advanced practitioner qualification of district and community nurse leaders.

Factor/item	No advanced qualification – (*n* = 107) (Mean ± *SD*)	Advanced qualification <2010 (*n* = 60) (Mean ± *SD*)	Advanced qualification ≥2010 (*n* = 281) (Mean ± *SD*)	*F*	*p*‐Value	Cohen's *d* (No qualification vs. <2010)	Cohen's *d* (No qualification vs ≥2010)
**Factor 1: Clinical care**	4.69 ± 0.51	4.69 ± 0.52	4.68 ± 0.53	0.447	0.640	0.00	−0.02
1. Take a holistic history	4.63 ± 0.67	4.48 ± 0.85	4.45 ± 0.86	1.839	0.160	0.20	0.22
10. Provide expert nursing care	4.75 ± 0.58	4.73 ± 0.66	4.72 ± 0.62	0.066	0.937	0.03	0.05
11. Manage clinical risk	4.79 ± 0.49	4.82 ± 0.47	4.79 ± 0.46	0.063	0.939	−0.06	0.00
15. Develop person‐centred relationship	4.70 ± 0.59	4.62 ± 0.76	4.62 ± 0.72	0.61	0.544	0.12	0.12
16. Support decisions about care	4.73 ± 0.54	4.73 ± 0.66	4.68 ± 0.66	0.387	0.679	0.00	0.08
17. Empower decisions about health	4.74 ± 0.57	4.68 ± 0.70	4.67 ± 0.67	0.442	0.643	0.10	0.11
18. Use evidence in clinical practice	4.78 ± 0.50	4.73 ± 0.55	4.80 ± 0.50	0.524	0.592	0.10	−0.04
**Factor 2: Leading practice**	4.29 ± 0.61	4.30 ± 0.58	4.29 ± 0.60	0.06	0.942	−0.02	0.00
33. Support professional development	4.70 ± 0.62	4.87 ± 0.39	4.79 ± 0.49	2.219	0.110	−0.34	−0.17
34. Devise learning programmes	4.18 ± 1.03	4.10 ± 1.08	4.04 ± 1.07	0.671	0.512	0.08	0.13
35. Mentor other staff	4.66 ± 0.57	4.75 ± 0.51	4.73 ± 0.58	0.607	0.545	−0.17	−0.12
36. Lead clinical practice	4.47 ± 0.76	4.62 ± 0.67	4.56 ± 0.70	1.03	0.358	−0.21	−0.13
37. Coordinate team	4.77 ± 0.51	4.85 ± 0.40	4.80 ± 0.43	0.672	0.511	−0.18	−0.06
40. Influence health policy	3.51 ± 1.20	3.60 ± 1.22	3.49 ± 1.23	0.183	0.832	−0.07	0.02
41. Lead service changes	3.63 ± 1.24	3.35 ± 1.27	3.64 ± 1.19	1.434	0.239	0.22	−0.01
**Factor 3: Clinical reasoning**	3.29 ± 0.98	3.31 ± 1.05	3.29 ± 0.98	0.014	0.986	−0.02	0.00
2. Physically examine health problems	3.99 ± 0.99	3.78 ± 1.14	3.84 ± 1.06	1.025	0.360	0.20	0.14
3. Order diagnostic tests	3.36 ± 1.09	3.13 ± 1.25	3.27 ± 1.14	0.783	0.458	0.20	0.08
4. Interpret diagnostic test results	3.12 ± 1.15	3.00 ± 1.21	2.98 ± 1.14	0.575	0.563	0.10	0.12
5. Develop differential diagnosis	3.14 ± 1.15	3.27 ± 1.23	3.19 ± 1.22	0.212	0.809	−0.11	−0.04
7. Prescribe medication	2.79 ± 1.58	3.35 ± 1.49	3.16 ± 1.34	3.794	0.023^*^	−0.37	−0.25
**Factor 4: Health promotion**	4.32 ± 0.73	4.22 ± 0.73	4.31 ± 0.75	1.044	0.353	0.14	0.01
25. Empower individuals to change behaviour	4.34 ± 0.80	4.20 ± 0.80	4.26 ± 0.87	0.578	0.562	0.18	0.10
26. Educate individuals about condition	4.60 ± 0.64	4.50 ± 0.70	4.53 ± 0.72	0.467	0.627	0.15	0.10
27. Coach for self‐management	4.44 ± 0.76	4.18 ± 0.81	4.37 ± 0.85	1.902	0.150	0.33	0.08
28. Educate about disease prevention	4.30 ± 0.91	4.12 ± 0.85	4.17 ± 0.98	0.944	0.390	0.20	0.14
29. Promote prevention in the community	4.28 ± 0.90	4.12 ± 1.08	4.21 ± 0.96	0.564	0.569	0.16	0.07
**Factor 5: Ethics**	4.48 ± 0.66	4.44 ± 0.77	4.47 ± 0.66	0.045	0.956	0.06	0.02
21. Use ethical reasoning	4.50 ± 0.65	4.42 ± 0.83	4.48 ± 0.70	0.309	0.734	0.11	0.03
22. Address ethical dilemmas	4.49 ± 0.68	4.50 ± 0.79	4.51 ± 0.69	0.029	0.971	−0.01	−0.03
23. Support self‐determination	4.44 ± 0.73	4.42 ± 0.85	4.41 ± 0.77	0.073	0.930	0.03	0.04

* Statistically significant *p*‐values.

### 5.4 Comparison Between District Nurse Team Leaders and APNs From Family and Community Nursing

Given the lack of statistically significant differences among district nurses holding or not an advanced qualification and the mixed effect sizes in favour of those with an advanced qualification or those without it, the district nurse team leaders, regardless of their advanced qualification, and APNs working in family and community nursing were compared. This comparison revealed statistically significant differences across all factors, except for ‘Leading Practice’, which did not show a statistically significant difference with a negligible effect size (*t* = 1.172, *p* = 0.242, Cohen's *d* = 0.07). The effect size (Cohen's *d*) for the ‘Clinical Reasoning’ factor was very large, with a considerably higher mean for family and community nurses. All other factors showed small/medium effect sizes. Table 4 presents the detailed descriptive statistics, *t*‐test, and effect sizes of this comparison for each factor and item.

**TABLE 4:** *t*‐Test between district and community nurse leaders and family and community nurses.

Factor/item	District nurses (*n* = 448) (Mean ± *SD*)	Family and community nurses (*n* = 548) (Mean ± *SD*)	*t*	*p*‐Value	Cohen's *d*
**Factor 1: Clinical care**	4.69 ± 0.51	4.86 ± 0.28	−6.758	< 0.001	−0.43
1. Take a holistic history	4.50 ± 0.82	4.91 ± 0.30	−10.972	< 0.001	−0.70
10. Provide expert nursing care	4.73 ± 0.62	4.80 ± 0.47	−1.899	0.058	−0.12
11. Manage clinical risk	4.80 ± 0.47	4.88 ± 0.34	−3.389	< 0.001	−0.22
15. Develop person‐centred relationship	4.64 ± 0.70	4.83 ± 0.43	−5.302	< 0.001	−0.34
16. Support decisions about care	4.70 ± 0.63	4.84 ± 0.39	−4.553	< 0.001	−0.29
17. Empower decisions about health	4.69 ± 0.65	4.86 ± 0.35	−5.423	< 0.001	−0.35
18. Use evidence in clinical practice	4.79 ± 0.51	4.91 ± 0.28	−4.891	< 0.001	−0.31
**Factor 2: Leading practice**	4.29 ± 0.61	4.24 ± 0.71	1.172	0.242	0.07
33. Support professional development	4.78 ± 0.52	4.58 ± 0.66	5.287	< 0.001	0.34
34. Devise learning programmes	4.08 ± 1.06	4.11 ± 0.96	−0.482	0.630	−0.03
35. Mentor other staff	4.71 ± 0.57	4.42 ± 0.78	6.663	< 0.001	0.42
36. Lead clinical practice	4.55 ± 0.71	4.55 ± 0.69	0.028	0.978	0.00
37. Coordinate team	4.80 ± 0.45	4.26 ± 0.89	11.53	< 0.001	0.73
40. Influence health policy	3.51 ± 1.22	3.85 ± 1.07	−4.622	< 0.001	−0.29
41. Lead service changes	3.60 ± 1.21	3.91 ± 1.06	−4.385	< 0.001	−0.28
**Factor 3: Clinical reasoning**	3.29 ± 0.98	4.62 ± 0.51	−27.531	< 0.001	−1.75
2. Physically examine health problems	3.87 ± 1.05	4.74 ± 0.61	−16.247	< 0.001	−1.03
3. Order diagnostic tests	3.28 ± 1.15	4.48 ± 0.77	−19.759	< 0.001	−1.26
4. Interpret diagnostic test results	3.02 ± 1.15	4.43 ± 0.77	−23.075	< 0.001	−1.47
5. Develop differential diagnosis	3.19 ± 1.20	4.76 ± 0.49	−27.939	< 0.001	−1.78
7. Prescribe medication	3.10 ± 1.43	4.69 ± 0.65	−23.262	< 0.001	−1.48
**Factor 4: Health promotion**	4.32 ± 0.73	4.57 ± 0.54	−6.299	< 0.001	−0.40
25. Empower individuals to change behaviour	4.27 ± 0.84	4.61 ± 0.61	−7.331	< 0.001	−0.47
26. Educate individuals about condition	4.54 ± 0.70	4.80 ± 0.45	−6.857	< 0.001	−0.44
27. Coach for self‐management	4.36 ± 0.82	4.55 ± 0.65	−3.976	< 0.001	−0.25
28. Educate about disease prevention	4.19 ± 0.95	4.55 ± 0.67	−6.942	< 0.001	−0.44
29. Promote prevention in the community	4.22 ± 0.96	4.36 ± 0.83	−2.499	0.013	−0.16
**Factor 5: Ethics**	4.48 ± 0.71	4.67 ± 0.54	−4.8	< 0.001	−0.33
21. Use ethical reasoning	4.50 ± 0.70	4.60 ± 0.62	−2.397	0.017	−0.31
22. Address ethical dilemmas	4.49 ± 0.68	4.51 ± 0.69	−2.367	0.018	−0.15
23. Support self‐determination	4.42 ± 0.77	4.70 ± 0.52	−6.854	< 0.001	−0.44

## 6 Discussion

This study aimed to delineate the advanced practice domains of district nurse team leaders, comparing those who completed their qualification following the policy reforms of 2010 and those who qualified prior to this date. The results suggest that district nursing team leaders have scored in all domains of family and community advanced practice slightly lower than their advanced practice nurse counterparts. This is in keeping with district nursing team leaders practising in a similar way to clinical nurse specialists with an emphasis on leading a wider team and managing patients who are experiencing an exacerbation of an existing condition. While this study is focused on a national context, the results can be read at the international level, as they highlight the importance of experiential learning in the development of advanced competences. In particular, the analysis on the impact of the advanced practice qualification on the competences of district nursing team nurse leaders revealed no statistically significant differences across the factors, and, even when the effect size were in favour of the groups with an advanced practice qualification, those effects were small or negligible. This lack of statistical significance suggests that the acquisition of advanced practice competencies may not be solely reliant on formal qualification attainment but rather on experiential learning provided in the clinical setting (Figueiredo et al. 2022). This is in keeping with the QNI report exploring the capability and confidence of the total district nursing workforce to prevent unnecessary admission to hospital (QNI 2021), which found the workforce to be capable and confident to take on such roles. In this research, the only statistically significant difference was found in the ability to prescribe medication, where those who obtained their qualification before 2010 reported higher scores. However, these results might also be read by considering the longer clinical expertise of this group and that those who held an advanced practice qualification tend to also hold a prescribing qualification, as demonstrated in the sample description and comparison among the three categories. Moreover, the exposure to clinical practice, and specifically the number of days spent providing care, did not differ across the three groups, and this supports the role of experiential learning in developing advanced practice competencies.

These findings also align with Kolb's (2015) experiential learning theory, which underscores the role of learning through experience, reflection, and application in professional development. Studies have demonstrated that advanced nursing competencies, such as clinical reasoning, leadership, and ethical decision‐making, evolve through direct patient care, mentorship, and reflective practice rather than through formal education alone (Kerr and Macaskill 2020; Benner 2021). This suggests that workplace exposure and clinical experience are fundamental in shaping advanced competencies among nurse leaders, regardless of the period in which they obtained their qualifications. It is important to recognise that experiential learning cannot exist in isolation and that theory also plays a role in the development of advanced competences (Dillard et al. 2024). It is evident that experiential learning bridges the gap between theory and practice, enhancing an advanced practitioner's ability to reason, make decisions, as well as improving their self‐efficacy and confidence (Yang et al. 2024; Flowers et al. 2020). Whether an APN is educated via a formal advanced practice programme or a combination of continuing professional development modules and experiential learning, the role of experience cannot be underestimated. Kerr and Macaskill (2020) argue that advanced nurse practitioners undergo role transition as they move from their existing registered nurse role to practising at a more advanced level. They argue that Benner's (2021) seminal work *From Novice to Expert* is less helpful for this transition, as the individual is probably never really a novice because of their previous experience. In addition, Benner's work does not really consider the nature of these roles, which require close integration into the healthcare system. Wood (1999) argues that for APNs, there are three stages of a transitional process which involve moving from idealism, where an individual has an idealised version of the role, through a process of organisational governance. This involves negotiating the role and its boundaries until the final stage of resolution is reached. The experiential learning process, with its cycles of learning through doing and reflection, is central to this role transition process.

Furthermore, credentialing has been emphasised as a crucial policy mechanism to acknowledge the tacit expert competence developed through experience. Credentialing frameworks serve as a structured approach to validate and recognise advanced practice competencies, particularly for practitioners who have accumulated extensive experiential learning (Royal College of Nursing 2020). A more structured and widespread credentialing system could mitigate the reliance on formal qualifications as the only indicator of expertise, ensuring that those with extensive clinical experience are recognised for their competencies. Credentialing is widely used in the United States for nurse practitioners as part of the process of licensure for advanced practice registered nurses (McMullen and Howe 2020). While in Ireland, a system known as a ‘development pathway’ enables individuals who have not followed a board‐approved programmes to gain APN registration (NMBI 2025). Despite the use of these routes, there is no evidence that evaluates the effectiveness of such routes in terms of ease of access to a licence to practice for those individuals who are already working as APNs prior to the implementation of the regulation.

As the secondary aim of this study, we compared district nurse team leaders with APNs working in family and community nursing services and highlighted significant differences across the factors, with APNs working in family and community nursing scoring higher in clinical care, clinical reasoning, health promotion, and ethics. This finding is consistent with the description of family and community nurses provided in Unsworth et al. (2025), which indicates that these healthcare professionals engage more frequently in autonomous decision‐making, diagnostic reasoning, and patient education. This is particularly evident in clinical reasoning, where the effect size indicated a very large effect in favour of family and community nurses. This is partly attributable to these APNs, spending a greater proportion of their time dealing with people with undifferentiated health problems than district nurse team leaders do. The Royal College of Nursing (2014) and the QNI (2024) suggest that district nursing team leaders spend around 40% of their time in direct care, with the remainder of the team spent leading services and managing a wider team. While statistical differences exist between the two groups, district nursing team leaders still performed many aspects of advanced practice nursing, such as history taking, physical examination, differential diagnosis, and prescribing to some extent, alongside aspects of clinical care, leading practice, health promotion, and ethics to a great extent. These differences are found in similar studies which have sought to delineate practice between different types of APNs. Jokiniemi et al. (2022b) examined the differentiation between nurse practitioners and clinical nurse specialists in Finland. The team found that clinical nurse specialists spent less time on comprehensive clinical care and spent more time on system support and education. A study in New Zealand also confirmed that clinical nurse specialists spent less time on direct clinical care than nurse practitioners (Carryer et al. 2018). Similar findings were reported by Oddsdóttir and Sveinsdóttir (2011) using daily activity diaries among clinical nurse specialists, where the greatest proportion of activity was related to education rather than direct clinical care.

Moreover, in this study, APNs working in family and community nursing demonstrated higher exposure to clinical practice compared to district nursing team leaders. In this vein, the higher scores in advanced competencies are consistent with the theoretical framework of experiential learning in clinical practice (Kolb 2015; Benner 2021).

A relevant difference was observed in clinical reasoning, where APNs working in family and community nursing demonstrated substantially higher scores compared to district nursing team leaders. This can be attributed to the expanded scope of practice for advanced nurse practitioners in family and community nursing, who often work in settings where they see people with undifferentiated health problems. Therefore, it requires independent diagnostic decision‐making and treatment planning (Unsworth et al. 2025). Their roles are designed to offer access to health services for people with acute undifferentiated diagnosis, emphasising advanced clinical assessment, differential diagnosis, and prescribing rights, which are critical components of their practice. In contrast, district nursing team leaders are often dealing with established health problems and exacerbations of these conditions.

Similarly, the significant difference in health promotion indicates that APNs working in family and community nursing engage more frequently in preventive care and patient education. This aligns with previous findings that highlight the role of APNs in family and community nursing in empowering individuals, coaching for self‐management and promoting public health initiatives (Unsworth et al. 2025). This distinction underscores the differentiated focus between district nurse team leaders who primarily manage care coordination and service leadership and APNs working in family and community nursing, who engage in more direct patient interaction and preventive interventions for newly diagnosed conditions.

Interestingly, leading practice did not show a statistically significant difference, suggesting that both groups engage in leadership roles at comparable levels. This finding may reflect the increasingly integrated nature of leadership responsibilities within advanced practice nursing, where both district nurse team leaders and APNs contribute to mentoring, team coordination, and influencing health policy.

## 7 Study Limitations

This study has several limitations; as a cross‐sectional study, it is not possible to generalise the findings to the wider population of district nurses. Moreover, future research should consider a longitudinal design in order to explore the determinants of advanced competencies among district nurses and family and community nurses. A longitudinal design could also disclose the evolutionary patterns of advanced competencies in nursing. To some extent, there could be an element of self‐selection bias with individuals who consider they are practising at an advanced level being more likely to respond to the survey. Furthermore, the online survey data collection strategy did not allow the estimation of the response rate. In addition, the Family and Community Nursing Advanced Practice Scale used in this study requires participants to estimate the extent to which each aspect of practice is performed in their role. The reliability of such self‐reported measures is subject to desirability bias and issues with memory and recall.

In terms of the secondary aim of the study, we compared a heterogeneous sample of district nurse team leaders with different qualifications against a sample where all of the participants have a recognised advanced practice nursing qualification, which may account for the variance in domain scores identified. However, the results of this comparison are useful in determining the extent to which district nursing team leaders have comparable levels of practice across the domains of advanced practice within a community nursing context.

## 8 Conclusion

This study explored the advanced practice competencies of district nurse team leaders, providing insights into the impact of policy reforms on their roles and competencies. The results indicate that formal qualifications alone do not determine the level of advanced practice competency; rather, experiential learning and clinical exposure play a crucial role in shaping district nurses’ advanced practice skills. This aligns with professional development theories, which highlight the importance of hands‐on practice in competency acquisition. Moreover, this highlights the need for structured credentialing frameworks to recognise the advanced competencies developed through clinical experience. Policymakers should consider enhancing experiential learning opportunities to ensure district nurses are adequately prepared to meet the demands of advanced practice roles.

## 9 Implications for Nursing and Health Policy

Policymakers should ensure that opportunities to develop skills and capability using experiential learning opportunities are available to the workforce and that such approaches are factored into regulatory systems. Planning structured pathways to enhance advanced practice competencies in clinical settings, combined with clear credentialing criteria, is key to recognising advanced practice nursing. Organisational settings, nursing regulatory bodies, and educational institutions should cooperate in identifying those criteria, ensuring that nurses can be formally acknowledged for skills acquired through clinical experience. Establishing national and international credentialing standards, based on validated and updated tools, is a key priority for the future of nursing care and healthcare systems’ sustainability. With the growing demands of care in managing long‐term conditions in community settings, policies should enable district nurses to work with greater autonomy, as this can reduce hospital admissions and improve patient outcomes. Investing in advanced postgraduate education, practice‐based mentorship programmes, and continuous professional development opportunities is crucial to implementing these policies, enhancing advanced practice, and improving patient outcomes.

## Author Contributions

Conceptualisation: JU, MT, GC, DC, VS, KM, JA, and CO. Methodology: JU, MT, GC, DC, VS, KM, and JA. Validation: JU and MT. Data curation: JU, CO, and MT. Writing—Original: JU, MT, KM, GC, VS, and DC. Writing—Review and Edit: JU, MT, GC, DC, VS, KM, JA, and CO.

## Conflicts of Interest

The authors declare no conflicts of interest.

## Use of Validated Tools

The authors have utilised the Family and Community Nursing—Advanced Practice Scale in this research. The authors are the copyright owners of the tool, and no permission was necessary to use it (Unsworth et al. 2025).

## Supporting information




**Supplementary File 1**: Comparison of sample characteristics across groups.
**Supplementary Table 2**: Comparison between District Nurses (DN) and Family and Community Nurses (FCN) characteristics.

Supporting Information
