# Supplementary material for: The Delineation of Advanced‐Level Practice Within UK District Nursing: A Cross‐Sectional Comparative Study Before and After Policy Implementation
Source: Int Nurs Rev. 2025 Aug 5;72(3):e70091. doi: 10.1111/inr.70091 (PMC12326113; doi:10.1111/inr.70091)
Supplement: Supplementary file 1 — Comparison of sample characteristics across groups. Supplementary Table 2: Comparison between District Nurses (DN) and Family and Community Nurses (FCN) characteristics. [file INR-72-0-s001.docx]

| Category | Subcategory (N,%) | No Advanced  Qualification | Advanced Qualification <2010 | Advanced Qualification ≥2010 | F / χ² | p-value |
| --- | --- | --- | --- | --- | --- | --- |
| Age | Mean ± SD | 46.60 ± 9.69 | 55.18 ± 6.42 | 43.69 ± 8.85 | 42.770 | < 0.001 |
| Gender | Female  (N, %) | 102 (95.3) | 60 (100.0) | 272 (96.7) | 2.788 | 0.248 |
|  | Male  (N, %) | 5 (4.7) | 0 (0.0) | 9 (3.3) |  |  |
| Highest Qualification | Degree  (N, %) | 63 (30.7) | 30 (14.6) | 112 (54.6) | 95.058 | < 0.001 |
|  | Diploma  (N, %) | 14 (100.0) | 0 (0.0) | 0 (0.0) |  |  |
|  | General Education (N, %) | 0 (0.0) | 1 (50.0) | 1 (50.0) |  |  |
|  | Masters  (N, %) | 14 (19.7) | 19 (26.8) | 38 (53.5) |  |  |
|  | Postgraduate Cert  (N, %) | 5 (15.2) | 6 (12.1) | 24 (72.7) |  |  |
|  | Postgraduate Diploma  (N, %) | 11 (8.9) | 6 (4.9) | 106 (86.2) |  |  |
| Holds a Prescribing Qualification | Yes  (N, %) | 56 (14.1) | 60 (15.2) | 280 (70.7) | 178.133 | < 0.001 |
|  | No  (N, %) | 51 (98.1) | 0 (0.0) | 1 (1.9) |  |  |
| Days per Week Providing Care | 1 day  (N, %) | 9 (16.1) | 9 (16.1) | 38 (67.9) | 18.063 | 0.054 |
|  | 2 days  (N, %) | 14 (30.4) | 4 (8.7) | 28 (60.9) |  |  |
|  | 3 days  (N, %) | 26 (30.6) | 19 (22.4) | 40 (47.1) |  |  |
|  | 4 days  (N, %) | 21 (18.6) | 14 (12.4) | 78 (69.0) |  |  |
|  | 5 days  (N, %) | 32 (25.8) | 12 (9.7) | 80 (64.5) |  |  |
|  | More than 5 days  (N, %) | 5 (20.8) | 2 (8.3) | 17 (70.8) |  |  |

Supplementary file 1. Comparison of sample characteristics across groups
