# Supplementary material for: The Delineation of Advanced‐Level Practice Within UK District Nursing: A Cross‐Sectional Comparative Study Before and After Policy Implementation
Source: Int Nurs Rev. 2025 Aug 5;72(3):e70091. doi: 10.1111/inr.70091 (PMC12326113; doi:10.1111/inr.70091)
Supplement: Supplementary file 2 — Supporting Information [file INR-72-0-s002.docx]

| Sample Characteristic | District Nurses | Family and Community Nurses | t / χ² | p-value |
| --- | --- | --- | --- | --- |
| Age (Mean ± SD) | 45.92 ± 9.57 | 50.12 ± 8.53 | -7.316 | < 0.001 |
| Days per Week Providing Care |  |  | 18.394 | 0.002 |
| 1 day (N,%) | 56 (62.2%) | 34 (37.8%) |  |  |
| 2 days (N,%) | 46 (41.1%) | 66 (58.9%) |  |  |
| 3 days (N,%) | 113 (39.9%) | 164 (60.1%) |  |  |
| 4 days (N,%) | 124 (40.8%) | 137 (59.2%) |  |  |
| 5 days (N,%) | 448 (45.0%) | 548 (55.0%) |  |  |
| More than 5 days (N,%) | 24 (55.8%) | 19 (44.2%) |  |  |

Supplementary table 2. Comparison between District Nurses (DN) and Family and Community Nurses (FCN) characteristics.
